# Comparative Efficacy of Intramuscular and Intradermal Porcilis Lawsonia^®^ Administration Under a Laboratory Challenge Model and Korean Field Conditions

**DOI:** 10.3390/vaccines14090803

**Published:** 2026-09-11

**Authors:** Sungseok Lee, Jincheol Kim, Sohyun Seok, Sangwon Lee, Sunsook Hwang, Jinyoung Hwang, Leonardo Domingo Ellerma, Chang-Seon Song

**Affiliations:** 1 KHAV Co., Ltd., 7th Floor, 120 Neungdong-ro, Gwangjin-gu, Seoul 05029, Republic of Korea; sungseoklee@khav.co.kr (S.L.); jc8229@khav.co.kr (J.K.); thgus8740@khav.co.kr (S.S.); 2 College of Veterinary Medicine, Konkuk University, 120 Neungdong-ro, Gwangjin-gu, Seoul 05029, Republic of Korea; odssey@konkuk.ac.kr; 3 Department of Immunology, School of Medicine, Konkuk University, Chungju 27478, Republic of Korea; sunhwang@kku.ac.kr; 4 MSD AH Korea Ltd., 22Fl., Seoul Square Building 416, Hangang-daero, Jung-gu, Seoul 04637, Republic of Korea; jinyoung.hwang@msd.com; 5 MSD Animal Health (Phils) Inc., 8767 Paseo De Roxas, 26/F AIA Tower, Makati 1200, Philippines; leonardo.ellerma@msd.com

**Keywords:** *Lawsonia intracellularis*, porcine proliferative enteropathy, intradermal vaccination, intramuscular vaccination, inactivated vaccine, vaccine efficacy, growth performance

## Abstract

**Background/Objectives**: *Lawsonia intracellularis* is a major enteric pathogen of swine that causes proliferative enteropathy, reduced growth performance, and economic losses. Although vaccination is an effective control strategy, comparative data on the efficacy of intramuscular (IM) and intradermal (ID) administration of inactivated *L. intracellularis* vaccines are limited. This study evaluated and compared the efficacy of Porcilis Lawsonia^®^ administered by IM or ID routes under experimental challenge and Korean field conditions. **Methods**: In a laboratory challenge study, 24 *L. intracellularis*-negative piglets were assigned to IM-vaccinated, ID-vaccinated, or unvaccinated control groups (*n* = 8/group). Vaccinated pigs received Porcilis Lawsonia^®^ and were orally challenged with *L. intracellularis* four weeks later. Clinical signs, average daily gain (ADG), antibody responses, bacterial shedding, and intestinal lesions were evaluated. In a field trial, 90 piglets from three commercial Korean farms were allocated to IM, ID, or control groups (n = 30/group) and monitored for growth performance, serological responses, and fecal shedding. **Results**: Under challenge conditions, both IM and ID vaccination significantly reduced clinical signs and fecal shedding while improving ADG and final body weight compared with controls. Vaccinated pigs developed significantly higher antibody responses before challenge. In field trials, both vaccination routes significantly increased body weight at 19 weeks of age and markedly reduced *L. intracellularis* shedding. No vaccine-related adverse effects were observed. Differences between IM and ID vaccination were generally not significant, although IM vaccination induced slightly higher antibody responses at 13 weeks of age under field conditions. **Conclusions**: Porcilis Lawsonia^®^ administered by either IM or ID routes provided comparable protective efficacy against *L. intracellularis* infection, reducing bacterial shedding and improving growth performance under both experimental and field conditions. These findings support ID vaccination as an effective alternative to conventional IM administration.

## 1. Introduction

*Lawsonia intracellualris* (*L. intracellularis*) is a Gram-negative, obligate intracellular bacterium which can infect wide a variety of hosts including the domestic and wild animal populations [1,2]. The infection of *L. intracellularis* could produce two forms of clinical presentation. Acute infection of *L. intracellularis* is described as proliferative hemorrhagic enteritis, characterized as diarrhea with hemorrhage and mortality of host. Another form of *L. intracellularis* infection, chronic infection of *L. intracellularis*, is known as porcine proliferative enteropathy (PPE). The chronic infection of *L. intracellularis* caused diarrhea, malnutrition, inflammation at the ileum and thickening of ileum and colon and decreased average weight gain (ADG) [3].

*L. intracellularis* is highly endemic pathogen worldwide. Several studies reported a high detection rate of *L. intracellularis* DNA from fecal samples, and serological investigation studies determined nearly 70–100% of pig herds were infected with *L. intracellularis* [4,5,6]. The economic loss due to the direct *L. intracellularis* infection was estimated at more than $1.63 USD [7]. However, in addition to the direct *L. intracellularis* infection, subclinical *L. intracellularis* infection often resulted in the retarded growth of pigs, high burden of medical intervention and prolonged market age. The total economic loss caused by the *L. intracellularis* infection was estimated about $5.84 USD ($8.33 AUD) [8].

Antibiotics are one of the first choices for the control of *L. intracellularis* infection. Several antimicrobics are known to be sensitive against *L. intracellularis*; however, there were differences between the sensitivity of antimicrobial drugs depending on the geographical location [9,10]. Antibiotic treatment against *L. intracellularis* infection often requires a continuous medication program, and prolonged use of antibiotics may lead to the emergence of antibiotic resistance of another enteric bacterium. Furthermore, the reduction of prophylactic antibiotics is required for public health concerns [11]. An alternative method such as vaccination is an attractive choice for the control of *L. intracellularis* infection. There are several commercially available vaccines including live-attenuated and killed vaccines. Several studies demonstrated vaccination against *L. intracellularis* infection could improve weight gain, reduced fecal shedding of *L. intracellularis* and lowered host mortality [12,13,14]. The exact mechanism of immune response by vaccination or natural infection of *L. intracellularis* is not yet fully understood. The absence of Immunoglobulin G (IgG) response or having a low level of cell-mediated immunity was not related to a lack of protection [15].

The vaccine antigen can be delivered by several routes. Intradermal injections offer time saving and better animal welfare over traditional intramuscular (IM) injection [16]. Intradermal (ID) vaccination of Porcine Circovirus type 2 (PCV2) and Porcine Reproductive and Respiratory Syndrome (PRRS) resulted in the early onset of cell-mediated immunity and shortened duration of vaccination procedure [17,18]. However, weak and ununiformed immune response was observed in the intradermal injection of Foot and Mouth (FMD) vaccine over intramuscular injection [19].

In this study, the efficacy of intradermal and intramuscular application of Porcilis Lawsonia^®^ was compared under a laboratory challenge model and Korean field conditions.

## 2. Materials and Methods

### 2.1. Study Design of Experimental Challenge Model

**Figure 1:**
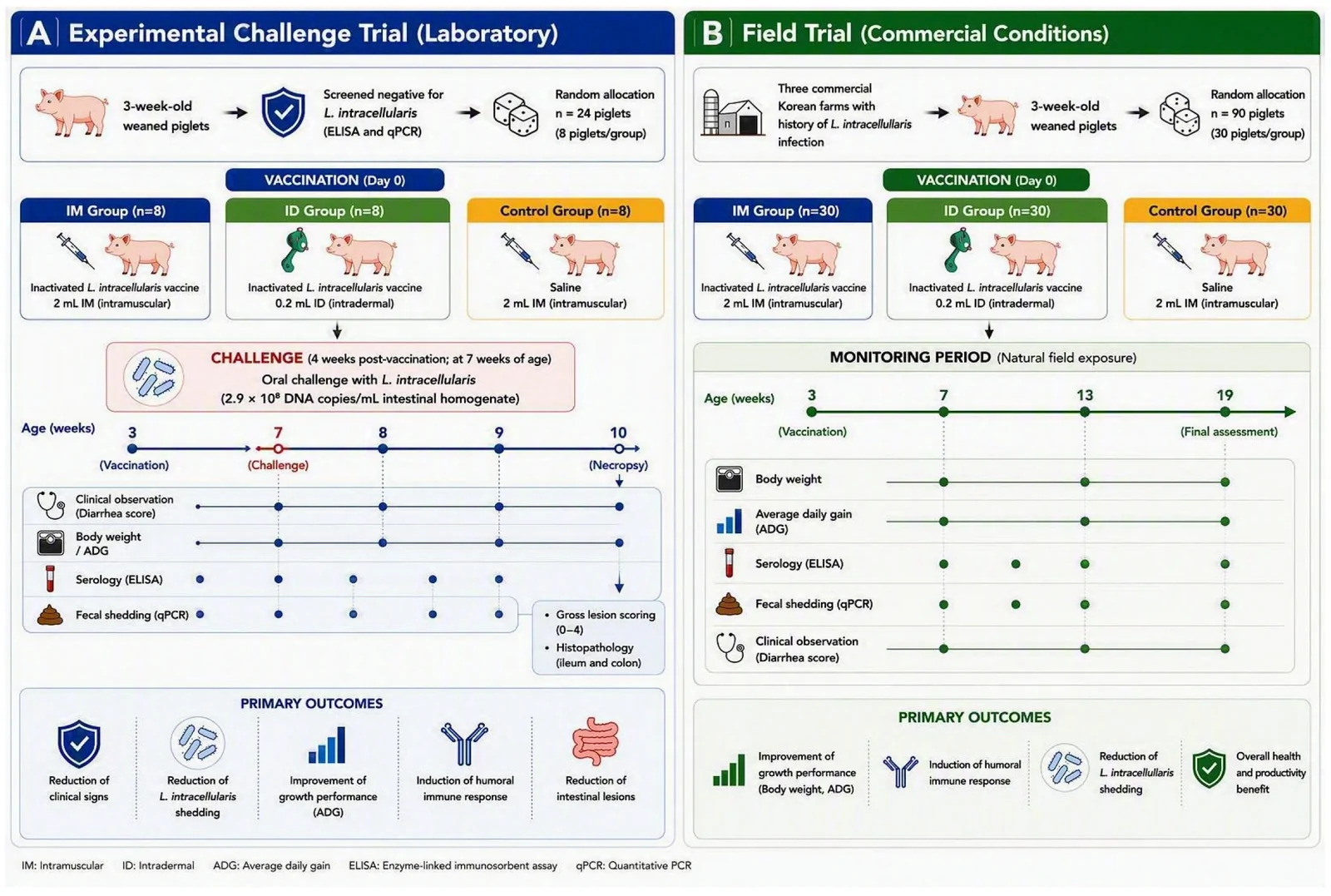
Experimental challenge and field-study design used to compare intramuscular (IM) and intradermal (ID) administration of an inactivated *Lawsonia intracellularis* vaccine. (**A**) Twenty-four piglets were allocated to IM-vaccinated, ID-vaccinated, or control groups and challenged orally with *L. intracellularis* four weeks after vaccination. Clinical observations, growth performance, serological responses, fecal shedding, and intestinal lesions were evaluated. (**B**) Ninety piglets from three commercial Korean farms were allocated to IM-vaccinated, ID-vaccinated, or control groups and monitored for growth performance, humoral immune responses, and fecal shedding under natural field exposure conditions.

Clinically healthy, 3-week-old piglets were purchased from a commercial pig farm with no history of clinical *L. intracellularis* outbreak for this trial. The absence of the *L. intracellularis*-specific antibody and antigen from the piglets was confirmed by *L. intracellularis* specific ELISA (SVANOVIR^®^ *L. intracellularis*/Ileitis-Ab, Indical bioscience GmbH, Svanova, Uppsala, Sweden) and quantitative PCR assay [20]. In total, 24 piglets were used for this study and divided into the Porcilis IM group (IM vaccinated, *n* = 8), Porcilis ID group (ID vaccinated, *n* = 8) and negative control (*n* = 8) (Figure 1A). All piglets were housed at different rooms during the entire study period. All piglets were ear-tagged and feed and water were provided ad libitum.

### 2.2. Vaccination and Challenge

All piglets except the negative control (*n* = 8) were vaccinated with Porcilis Lawsonia^®^ according to the instruction of manufacturer. The Porcilis IM group received 2 mL of vaccine via the intramuscular route and 0.2 mL of vaccine was administered by the IDAL™ vaccine device via intradermal route for the Porcilis ID group. The negative control group received 2 mL of normal saline via intramuscular route.

For the challenge trial, intestinal samples were collected from the recent *L. intracellularis* outbreak and homogenized. The homogenate was inoculated into naïve pigs to validate reproducibility of *L. intracellularis* infection. More than half of the pigs showed clinical signs of *L. intracellularis* infection and intestinal homogenate was assumed to reproduce clinical *L. intracellularis* infection after inoculation and used for the trial. All piglets were challenged orally with 20 mL of *L. intracellularis*-infected intestinal homogenate at 7 weeks of age (4 weeks post-vaccination). The homogenate had about 2.9 × 10^8^ copies of *L. intracellularis* DNA per 1 mL.

### 2.3. Clinical Evaluation of Pigs

All pigs in this trial were monitored during the entire periods to evaluate and compare the efficacy of Porcilis Lawsonia^®^ with different administration methods and an unvaccinated control group. Heathy pigs were scored as 0, pigs with ruffled hair were as 1, mild diarrheic pigs were 2, severe or blood stool was 3 and mortality was recorded as 5, respectively.

### 2.4. Gross Pathology and Microscopic Lesion Evaluation

At the end of this trial, all pigs were humanely euthanized by electric stunning and bleeding, then necropsy was performed. During necropsy, gross lesion was scored as severe inflammation/thickening of intestinal wall and/or hemorrhagic lesion = 4, severe intestinal thickening = 3, moderate = 2, mild = 1 slight = 0.5 and normal = 0 as previously described [21]. Furthermore, the small intestine (duodenum), ileum and large intestine (cecum) were collected and stained with Hematoxylin–Eosin (HE) for microscopic lesion evaluation. Lesion was scored from 0 (normal, no hyperplastic enterocytes) to 3 (hyperplastic crypts were present) as previously described [22].

### 2.5. Evaluation of Humoral Immune Response

At 3 weeks of age (before vaccination), 7 weeks (before challenge), 8 weeks (1 week post-challenge, wpc), 9 weeks (2 wpc) and 10 weeks (3 wpc), serum samples were collected to determine the humoral immune response induced by vaccination or challenge. Serum samples were kept at −80 °C until use. After trial, humoral immune response was measured by a commercial ELISA kit.

### 2.6. Quantification of L. Intracellularis Shedding After Challenge

As with same time points of serum sample collection, fecal samples were also collected from individual pigs and kept at −80 °C until use. DNA was extracted from the fecal sample using the QIAamp Fast DNA Stool Mini Kit (QIAGEN GmbH, Hilden, Germany) according to the instruction. Quantitative PCR was carried out as previously described [20]. For the generation of standard curve analysis, the PCR product was inserted into the TA cloning vector and the DNA copy number was calculated based on the plasmid DNA base pair length.

### 2.7. Field Trial Design

For this study, three commercial pig farms with a history of *L. intracellularis* infection and/or clinical outbreak were selected. Serological investigation was performed to determine the age of infection and the results showed that pigs in the trial farms were infected by *L. intracellularis* about 13~19 weeks of age respectively. A total of ninety clinically healthy, three-week-old piglets were assigned for this trial. The Porcilis IM group (*n* = 30) was vaccinated by Porcilis Lawsonia^®^ via intramuscular route, the Porcilis ID group (*n* = 30) intradermally, and normal saline was injected intramuscularly for the negative control group (*n* = 30). All piglets were ear-tagged and housed separated but shared same treatment except vaccination in this trial (Figure 1B).

To determine the onset of immunity by vaccination and the effect of vaccination on the shedding of *L. intracellularis*, serum and fecal samples from ten pigs per groups were collected at 3, 7, 13 and 19 weeks of age and were kept at −80 °C until further process. All pigs were weighed individually at 3, 7 and 19 weeks of age to compare the productivity of pigs between groups.

### 2.8. Statistical Analysis

All results generated in this trial were statistically analyzed using GraphPad Prism (GraphPad, San Diego, CA, USA, software version 8.0). The results were considered significant when the *p* values were lower than 0.05 respectively. Statistical significance in humoral immune responses and lesion score post-challenge were examined using the Kruskal–Wallis test with Dunn’s multiple comparison test.

### 2.9. Ethical Statement

This study was performed by Konkuk Human and Animal Vaccine (KHAV, Seoul, Republic of Korea) Co., Ltd. and the use of animals and protocols were reviewed and approved by KHAV IACUC (Approval number IACUC-A25001).

## 3. Results

### 3.1. Experimental Challenge Results—Clinical Signs and Average Daily Gain (ADG)

**Figure 2:**
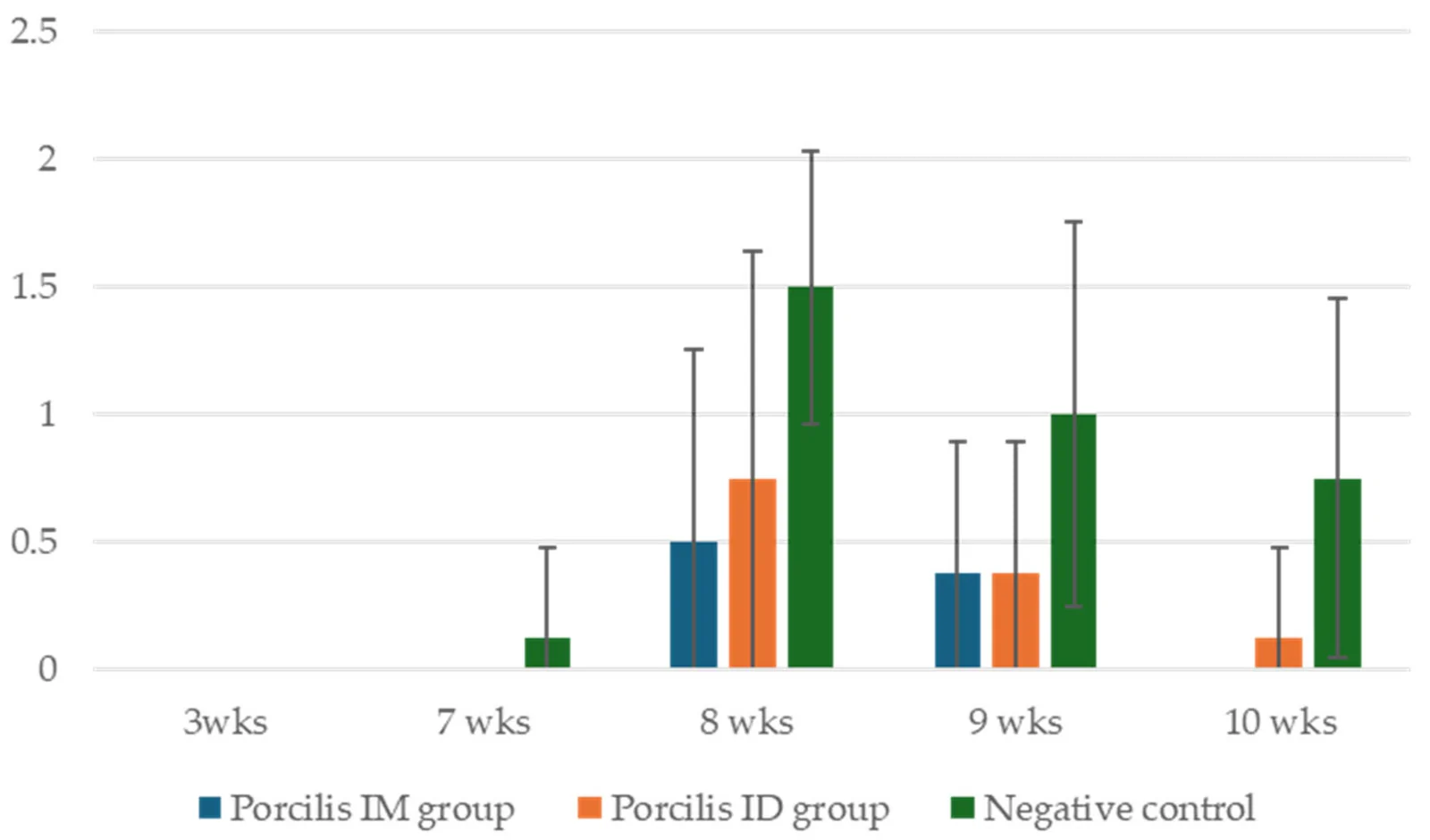
Clinical signs observation after vaccination and *L. intracellularis* challenge. All piglets in this trial were individually monitored and clinical signs were evaluated as 0 = Bright and alert; 1 = Ruffled hair and/or pale skin; 2 = Diarrhea; 4 = Bloody diarrhea, emaciation; and 6 = Mortality of host. The negative group exhibited signs of *L. intracellularis* infection; however, a significantly lower clinical score was recorded in both vaccinated groups.

Adverse effect such as swelling at the injection site, fever and shock was not present after vaccination at Porcilis IM- and ID-vaccinated group. Pigs in the negative control group developed signs of *L. intracellularis* infection (clinical signs of depression, ruffled hair and diarrhea) after about two weeks post-challenge. However, no bloody stool or mortality of the host was present in this trial. At 9 and 10 weeks of age, Both the IM- and ID-vaccinated groups showed statistically lower clinical scores compared to the negative control group (*p* value < 0.05), but there were no statistical differences between vaccine groups (Figure 2).

**Figure 3:**
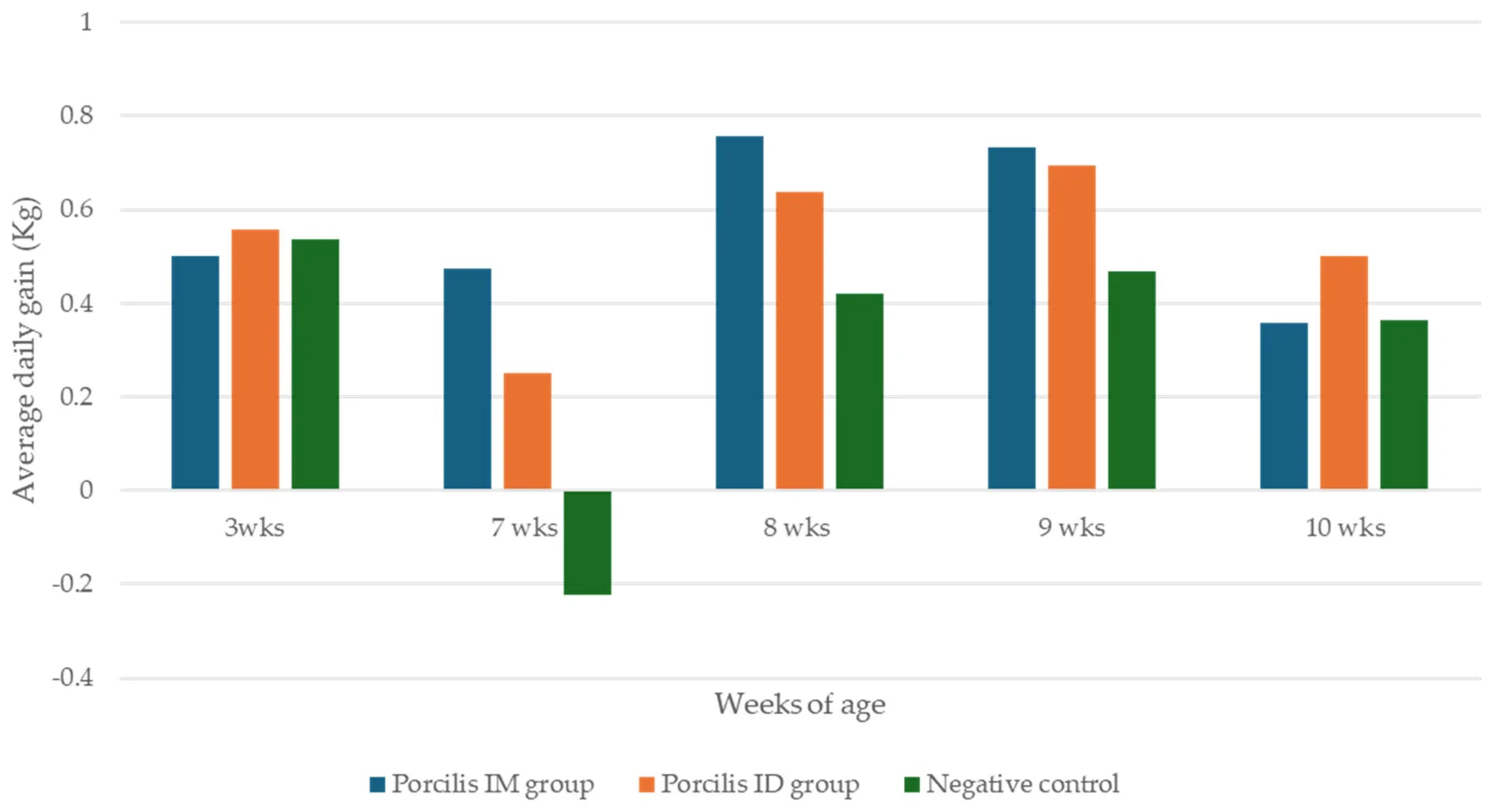
Productive performance evaluation after vaccination. Average daily gain (ADG) of pigs was evaluated to determine the effect of vaccination after challenge. ADG of Porcilis IM vaccinated group was 0.475 kg/day, 0.252 kg/day for Porcilis ID group and −0.221 kg/day for negative control group.

Average daily gain (ADG) was calculated with individual pig weight (Figure 3). After challenge, pigs in the negative control group showed −0.221 kg/day of weight reduction, suggesting successful challenge. However, pigs in both vaccinated groups gained weight and higher ADG compared to the negative control group (*p* value < 0.05), with no statistical difference between vaccine groups (*p* value > 0.3) being present. Both vaccinated groups had pigs of higher weights at the end of trial, with a significant difference (*p* value < 0.05). The average weight of the Porcilis IM vaccinated group at 10 weeks of age was 37.1 kg, ad 36.1 kg for the Porcilis ID vaccinated group, and 29.8 kg for the negative control group, respectively.

### 3.2. Experimental Challenge Results—Humoral Immune Response After Vaccination and Challenge

**Figure 4:**
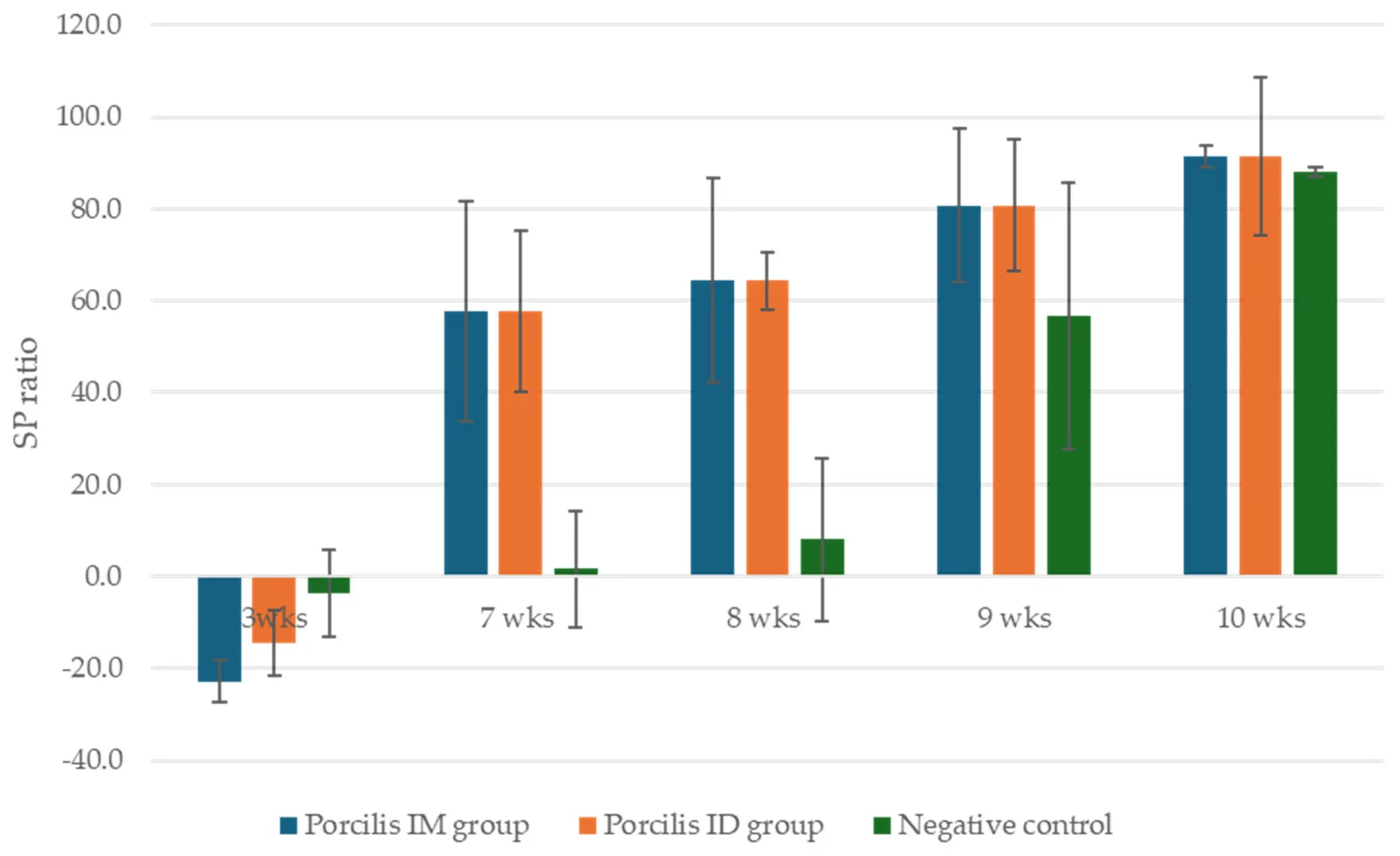
Determination of humoral immune response after vaccination and challenge. *L. intracellularis* specific IgG was quantified by ELISA assay. SP ratio was generated according to the instruction of ELISA kit. No piglets in this trial were seropositive against *L. intracellularis* at the beginning of the trial. Humoral immunity was developed after vaccination, about 75% of pigs were seropositive after 4 weeks post-vaccination (7 weeks of age) and 100% of them were seropositive at both vaccinated groups after 8 weeks of age.

Before vaccination, all piglets were negative for the *L. intracellularis*-specific antibody (Figure 4). After 4 weeks post-vaccination, both vaccinated groups (Porcilis IM and ID group) showed significantly high level of humoral immunity induced by vaccination compared to the negative control (*p* value < 0.001), but the level of humoral immunity was not different between vaccinated groups (*p* value = 0.13). The negative control developed antibody response after challenge, but the humoral immune response was lower compared to the vaccinated groups at 9 weeks of age significantly (*p* value < 0.001). Three weeks after challenge, all pigs in this trial were seropositive against *L. intracellularis* and the level of humoral immunity was not significantly different (*p* value > 0.05).

### 3.3. Experimental Challenge Results—Shedding of L. intracellularis After Challenge

**Figure 5:**
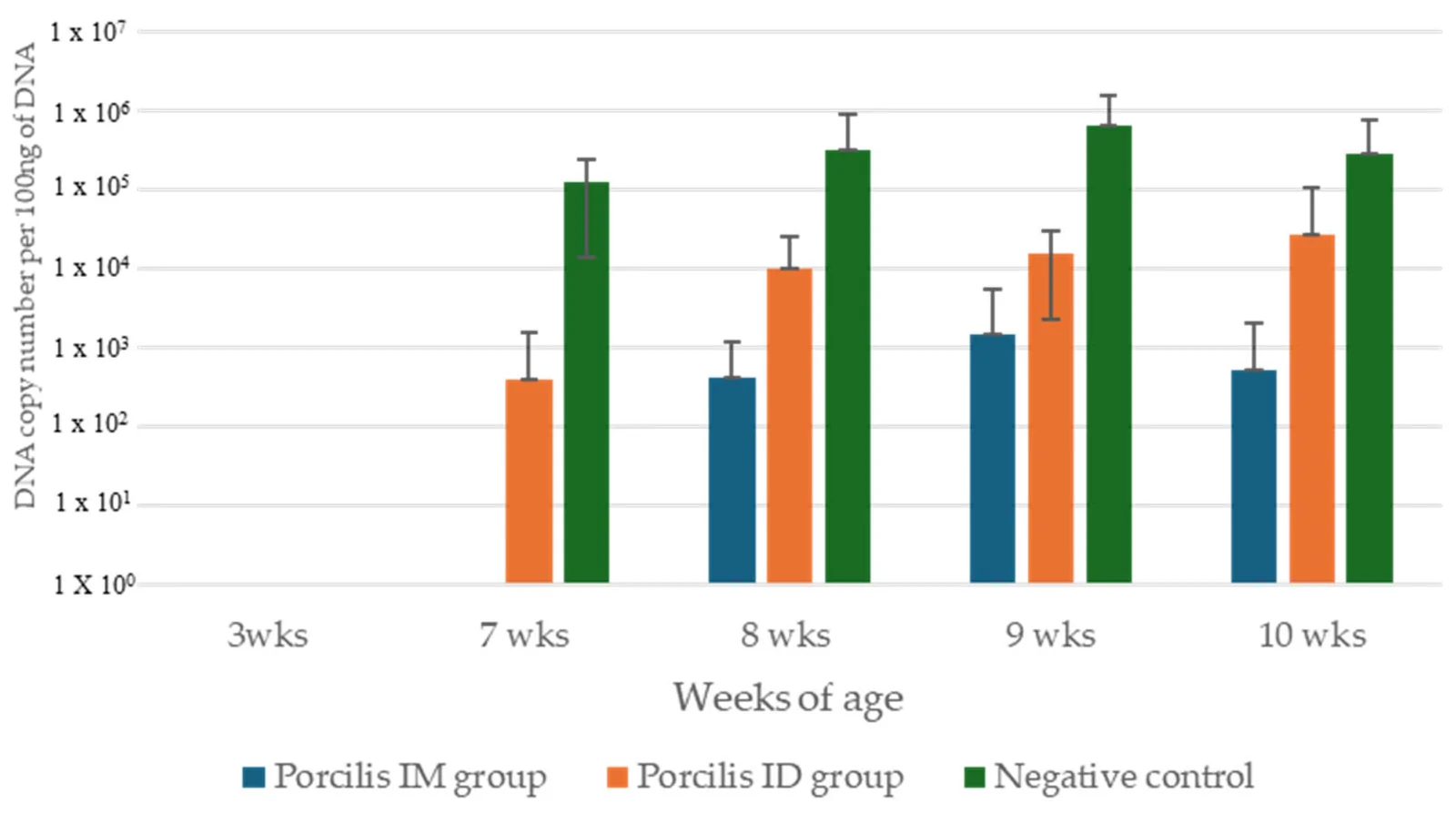
The level of *L. intracellularis* shedding after challenge. After challenge, the shedding of *L. intracellularis* was quantified using a quantitative PCR assay. The amount of *L. intracellularis* DNA was expressed as DNA copy number per 100 ng of total DNA. After challenge, the peak of shedding was observed 9 weeks of age (2 wpc). However significant reduction in *L. intracellularis* shedding was observed at both vaccinated groups (*p* value < 0.05) compared to the negative control group. The average of *L. intracellularis* shedding in the Porcilis IM group was lower than the Porcilis ID group but the difference was not significant (*p* value > 0.7).

Fecal samples from the individual pigs were evaluated to compare the amount of *L. intracellularis* shed after challenge (Figure 5). Both the IM- and ID-vaccinated groups exhibited significantly lower shedding of *L. intracellularis* during the entire trial period (*p* value < 0.05) compared to the negative control group respectively.

### 3.4. Experimental Challenge Results—Gross and Microscopic Lesion Score

**Figure 6:**
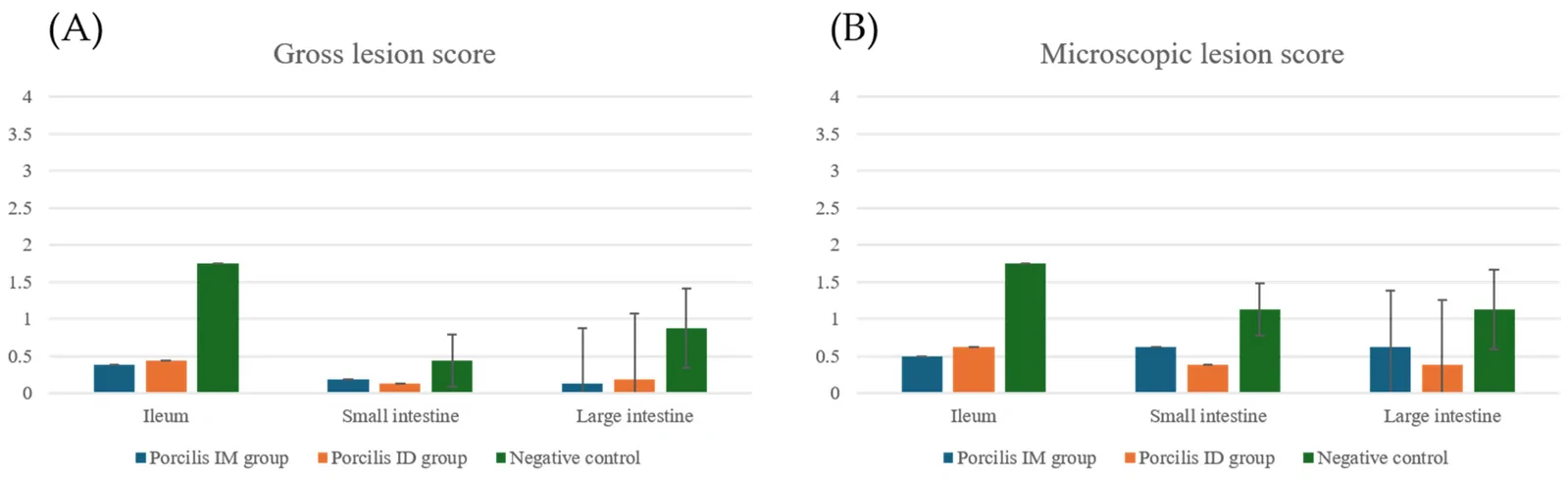
Evaluation of macroscopic and microscopic lesion. (**A**) The severity of macroscopic lesion in the small intestine, large intestine and ileum was visually inspected and scored during the necropsy. (**B**) The severity of microscopic lesion in the small intestine, large intestine and ileum was scored during the necropsy. A histopathological lesion was mainly found in the large intestinal system, especially the ileum. However, the severity of the lesion was lower in both vaccinated groups compared to the negative control group.

During necropsy, intestinal system was evaluated to determine the severity of *L. intracellularis* infection between groups (Figure 6). During the necropsy, the intestinal system was evaluated and scored. Both vaccine groups showed lower gross lesion scores compared to the negative control group, but the result was not significant. Intestinal tissue samples were further stained with Hematoxylin–Eosin and scored to determine the protective efficacy of Porcilis Lawsonia^®^. Both vaccine groups had more than two times lower scores in the ileum and small intestine tissue; however, the result was not statically significant.

### 3.5. Field Trial Results—Growth Performance

**Figure 7:**
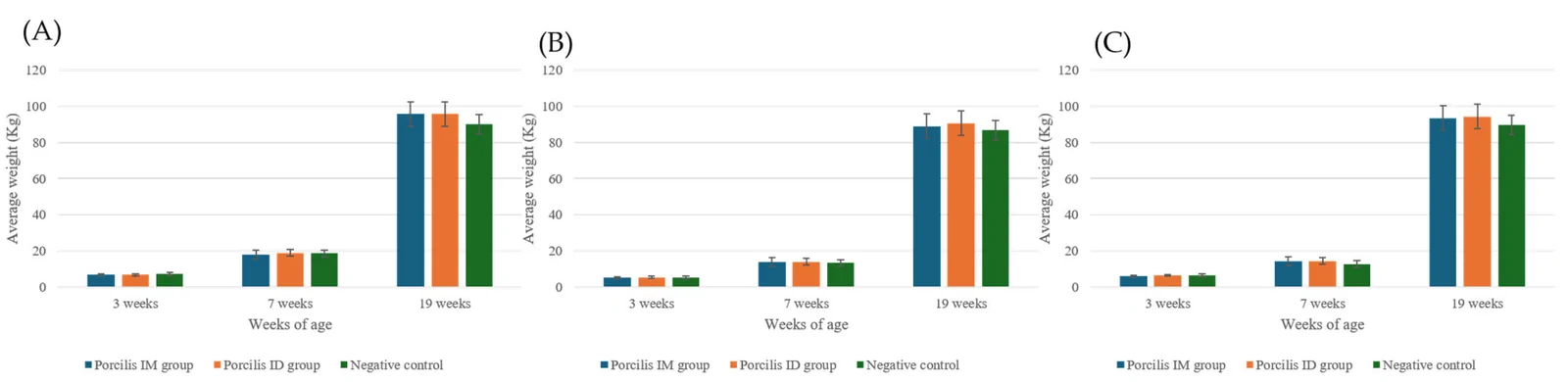
Growth performance between different groups. All pigs were individually weighed and average weight of the groups was compared. (**A**) Mean weight of Farm A. (**B**) Mean weight of Farm B. (**C**) Mean weight of Farm C. The difference in the average pig weight of the negative control group ranged from approximately 4.0~5.7 kg lower than both vaccinated groups. The average pig weight between the IM- and ID-vaccinated ranged 0.1~0.7 kg with no statistical significance (*p* value > 0.05).

No adverse effects such as swelling, shock and mortality were not observed in the piglets of three farms used in this study after vaccination. The growth performance between groups was evaluated by weighing all pigs individually to determine the effect of vaccination and delivery route (Figure 7). At the early ages of pigs (3 and 7 weeks of age), there was no statistical differences observed between the vaccinated groups and the negative control group (*p* value > 0.05) among the three trial farms. At 19 weeks of age, both vaccinated groups at the three trial farms showed significantly higher average weights compared to the negative control group (*p* value < 0.001). However, there was no significant different between the IM-vaccinated and ID-vaccinated groups across all three trial farms (*p* value > 0.05).

### 3.6. Field Trial Results—Humoral Immune Response After Vaccination

**Figure 8:**
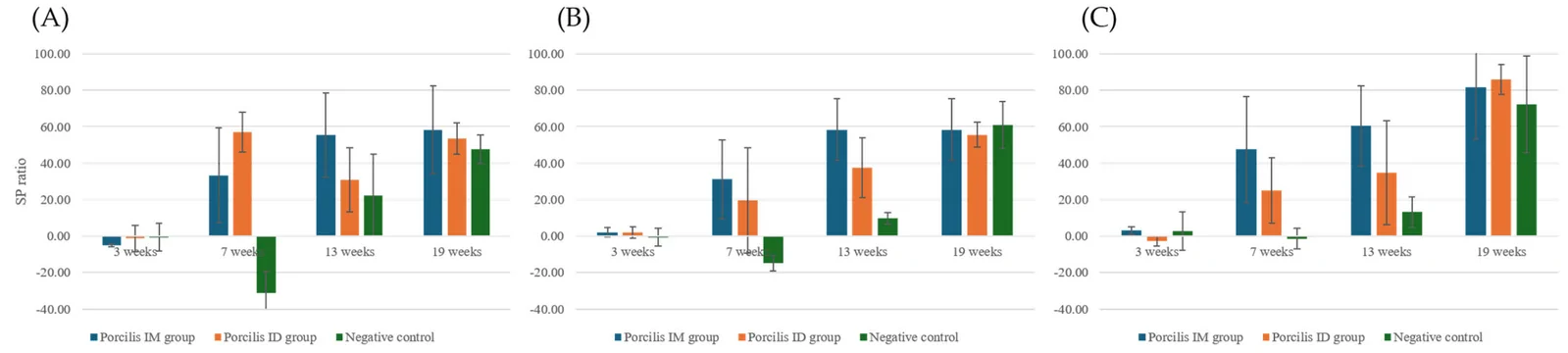
Detection of humoral immunity against *L. intracellularis* after vaccination and natural infection. (**A**) Mean SP ratio of Farm A. (**B**) Mean SP ratio of Farm B. (**C**) Mean SP ratio of Farm C. Ten pigs per group were randomly selected and serum samples were collected. An ELISA assay was carried out to determine humoral immunity elicited by vaccination or natural infection.

At 3 weeks of age, the mean SP ratio determined by the ELISA assay showed no significant difference among groups and farms (*p* value = 0.8416). After vaccination (7 weeks of age), a significant increase in the SP ratio in both the Porcilis IM and ID groups was observed compared to the negative control group (*p* value < 0.00001). At 13 weeks, the disparity between vaccinated groups and the negative control group remained highly significant (p<0.0001). A detailed post hoc Tukey HSD analysis revealed a statistically significant difference between the Porcilis IM and ID groups. The Porcilis IM group maintained a higher SP ratio compared to the Porcilis ID group (*p* value = 0.0207). After natural infection, no statistically significant differences were observed between the Porcilis IM and ID groups, nor between the vaccinated and negative control groups at 19 weeks of age (Figure 8).

### 3.7. Field Trial Results—Reduction in L. intracellularis Shedding After Vaccination

**Figure 9:**
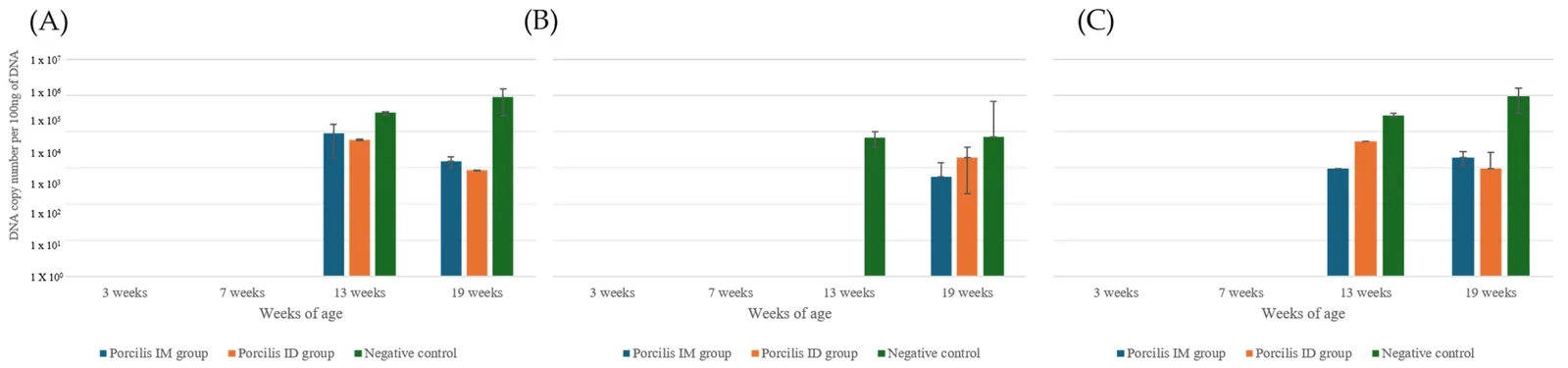
Reduction in *L. intracellularis* shedding after vaccination. Statistical comparison of the efficacy of Porcilis IM and Porcilis ID groups against the negative control group. (**A**) Comparison of *L. intracellularis* DNA between groups in Farm A. (**B**) Comparison of *L. intracellularis* DNA between groups in Farm B. (**C**) Comparison of *L. intracellularis* DNA between groups in Farm C.

At 3 and 7 weeks of age, *L. intracellularis* DNA was not detected across all groups. However, at 13 weeks, the negative control group exhibited a significant amount of *L. intracellularis* DNA copy numbers in the fecal samples, indicating the occurrence of subclinical *L. intracellularis* infection at the three trial farms (Figure 9). Both vaccinated groups successfully suppressed the shedding of *L. intracellularis*, resulting in highly significant differences among the groups (p<0.05 across all farms). By 19 weeks, the vaccinated groups consistently maintained substantially lower *L. intracellularis* shedding compared to the negative control group.

## 4. Discussion

*L. intracellularis* infection has caused tremendous economic losses world widely. Several antibiotics such as macrolides, tetracyclines, lincosamides and pleuromutilins via feed medication and drinking water showed efficacy for the control of *L. intracellularis* infection [16]. However, public health concerns about the antibiotic resistance and concept of One health hampered the use of prophylactic antibiotics, especially for farm animals [17]. Vaccination also has been implemented worldwide. The oral administration of a commercially available live-attenuated vaccine showed a significant improvement in productivity and protection against *L. intracellularis* infection under laboratory and field condition [18,19]. However, individual oral vaccine administration is labor-intensive and time-consuming. To address these limitations, an inactivated form of the *L. intracellularis* vaccine with intramuscular (IM) or intradermal (ID) administration was developed, and its efficacy was evaluated under laboratory and Korean field conditions.

Under experimental laboratory conditions, no vaccine-related adverse effects—such as fever, dyspnea, or shock—were observed in both IM- and ID-vaccinated pigs throughout the entire study period. Following the subsequent challenge with *L. intracellularis*, the negative control group exhibited a transient decrease in body weight, accompanied by clinical symptoms including fever (above 39.5 °C) and diarrhea in several pigs. However, these clinical signs were significantly suppressed in both the IM- and ID-vaccinated groups (*p* value < 0.05). And the vaccinated groups successfully prevented the acute decline in average daily gain (ADG) after *L. intracellularis* challenge compared to the negative control group (*p* value < 0.05), maintaining a significantly higher average body weight until the end of the study.

Regarding the pathological and immunological mechanisms, the vaccinated groups demonstrated a highly significant reduction in fecal shedding of *L. intracellularis* during the early-to-mid post-challenge period (7–9 weeks of age) compared to the negative control group (*p* value < 0.01). Furthermore, a significant seroconversion was already observed prior to the challenge at 8 weeks of age (*p* value < 0.01). In the Porcilis IM group, about 75% of piglets were seropositive against *L. intracellularis* at 4 weeks post-vaccination and 50% of seropositivity was observed in the Porcilis ID group. All vaccinated pigs were seroconverted after 5 weeks post-vaccination. Macroscopic and microscopic postmortem analysis also indicated the successful prevention of *L. intracellularis* infection in the intestinal system.

Under the Korean field condition, both IM and ID vaccination showed safety and a significant improvement in productivity. More than a fivefold reduction in *L. intracellularis* shedding and higher average weight were observed and the early market day indicated the successful protection of both IM and ID vaccination against subclinical *L. intracellularis* infection.

## 5. Conclusions

In conclusion, the results of this laboratory challenge model and Korean field condition trial indicate that the Porcilis Lawsonia^®^ vaccine induces effective protective immunity in pigs, providing robust defense against *L. intracellularis* challenge by alleviating clinical symptoms, reducing bacterial shedding, and improving growth performance. The precise mechanism of protection is not yet fully understood, however the humoral immune response and the level of protection induced by IM and ID administration of the vaccine was not significantly different in both the laboratory challenge model and field conditions. Needle-free intradermal vaccination may also contribute to improved animal welfare and hygiene in clinical practice.

## Data Availability

Data can be made available upon reasonable request to the corresponding author.
